# Sex Differences in Executive Functioning in the LEAP! Rx Exercise Study

**DOI:** 10.1093/geroni/igaf122.2751

**Published:** 2025-12-31

**Authors:** Gabrielle Lewis, Amanda Szabo-Reed, Jordan Baker, Jonathan Clutton, Mickeal Key, Jeff Burns, Amber Watts

**Affiliations:** University of Kansas, Lawrence, Kansas, United States; University of Kansas Medical Center, Kansas City, Kansas, United States; University of Kansas Medical Center, Kansas City, Kansas, United States; University of Kansas Medical Center, Kansas City, Kansas, United States; University of Kansas Medical Center, Kansas City, Kansas, United States; University of Kansas Medical Center, Kansas City, Kansas, United States; University of Kansas, Lawrence, Kansas, United States

## Abstract

Executive functioning is critical in maintaining independence in older adulthood. Women’s cognition tends to decline more rapidly. Previous research suggests aerobic exercise benefits executive functioning. It remains unclear whether sex moderates the cognitive benefit of exercise. We hypothesized that men and women would differ in executive function in response to the LEAP! Rx exercise intervention. We analyzed data from 219 older adults (M age=72.36) in the LEAP! Rx exercise intervention. We randomized participants to the intervention (n = 110) or control group (n = 109). We measured executive functioning using the Flanker Inhibitory Control and Attention Test (Flanker) and Dimensional Change Card Sort Test (DCCS). We conducted two 2-way repeated measures ANOVAs analyzing the effect of sex and group (intervention vs. control) on change in Flanker and DCCS scores across Baseline, 12 weeks, and 52 weeks, controlling for age and education. Results indicated no main effects of the intervention (p>.30), nor of sex (p>.40), at any time point. There was no significant interaction between sex and intervention group (p=.89). Previous results in this sample showed that although the intervention group significantly improved in cardiorespiratory fitness compared to the control group, the intervention did not appear to influence overall cognitive performance. Executive functioning scores had a ceiling effect, limiting our ability to detect improvements. Future research should include more challenging tests of executive function to investigate mechanisms by which exercise may improve executive function, such as cardiorespiratory fitness or intervention adherence. Our project contributes to examinations of individual characteristics most likely to benefit from exercise.

